# Development of an Enzyme-Linked Immunosorbent Assay for Rapid Detection of Dengue Virus (DENV) NS1 and Differentiation of DENV Serotypes during Early Infection

**DOI:** 10.1128/JCM.00221-19

**Published:** 2019-06-25

**Authors:** Szu-Chia Lai, Yu-Yine Huang, Pei-Yun Shu, Shu-Fen Chang, Po-Shiuan Hsieh, Jiunn-Jye Wey, Meng-Hung Tsai, Ren-Jy Ben, Yi-Ming Hsu, Yi-Chien Fang, Mei-Ling Hsiao, Chang-Chi Lin

**Affiliations:** a Institute of Preventive Medicine, National Defense Medical Center, Taipei City, Taiwan; b Taiwan Centers for Disease Control, Taipei City, Taiwan; c Kaohsiung Armed Forces General Hospital, Kaohsiung City, Taiwan; d Zuoying Branch of Kaohsiung Armed Forces General Hospital, Kaohsiung City, Taiwan; e Tangshan Branch of Kaohsiung Armed Forces General Hospital, Kaohsiung City, Taiwan; Rhode Island Hospital

**Keywords:** dengue, ELISA, NS1, clinical diagnosis, serotype, typing

## Abstract

Dengue fever, caused by infections with the dengue virus (DENV), affects nearly 400 million people globally every year. Early diagnosis and management can reduce the morbidity and mortality rates of severe forms of dengue disease as well as decrease the risk of wider outbreaks. Although the early diagnosis of dengue can be achieved using a number of commercial NS1 detection kits, none of these can differentiate among the four dengue virus serotypes. In this study, we developed an enzyme-linked immunosorbent assay (ELISA) for the detection of dengue virus (DENV) NS1 by pairing a serotype-cross-reactive monoclonal antibody (MAb) with one of four serotype-specific MAbs in order to facilitate the rapid detection of NS1 antigens and the simultaneous differentiation of DENV serotypes. A total of 146 serum samples obtained from patients suspected to be in the acute phase of DENV infection were used to evaluate the clinical application of our novel test for the detection and serotyping of DENV. The overall sensitivity rate of our test was 84.85%, and the sensitivity rates for serotyping were as follows: 88.2% (15/17) for DENV serotype 1 (DENV1), 94.7% (18/19) for DENV2, 75% (12/16) for DENV3, and 66.6% (6/9) for DENV4. Moreover, there was no cross-reactivity among serotypes, and no cross-reactivity was observed in sera from nondengue patients. Thus, our test not only enables the rapid detection of the dengue virus but also can distinguish among the specific serotypes during the early stages of infection. These results indicate that our ELISA for DENV NS1 is a convenient tool that may help elucidate the epidemiology of DENV outbreaks and facilitate the clinical management of DENV infections.

## Abstract (precis)

Dengue fever, caused by infections with the dengue virus (DENV), affects nearly 400 million people globally every year. Early diagnosis and management can reduce the morbidity and mortality rates of severe forms of dengue disease as well as decrease the risk of wider outbreaks.

## INTRODUCTION

Global climate change, growing populations, mass urbanization, and modernized transportation have expanded the distribution of the mosquito vectors, Aedes aegypti and Aedes albopictus, of dengue virus (DENV), resulting in dengue fever now being considered a global burden. More than half of the world’s population (3.6 billion) currently lives with the risk of dengue virus infection, and approximately 390 million people are infected with dengue virus every year, with 96 million of these infected individuals displaying symptoms of the disease (1–5), leading to approximately 20,000 deaths per 50 million to 100 million cases of dengue fever annually (6). Indeed, the prevalence of dengue fever poses a serious threat to human health and greatly impacts the economy. The annual global cost of dengue fever (direct and indirect) has been estimated to be $8.9 billion (7).

Dengue fever can be caused by any one of four pathogenic serotypes (DENV serotype 1 [DENV1] to DENV4). Infection with any serotype results in lifelong immunity to that serotype; however, this immunity provides only short-term cross-protection against the other serotypes (8, 9). Furthermore, the antibodies generated during the first infection can increase the likelihood that a second infection (with another serotype) will develop into a severe disease, dengue hemorrhagic fever or dengue shock syndrome (10–13). In some tropical regions, the simultaneous presence of multiple DENVs can lead to situations in which the disease is hyperendemic (i.e., the cocirculation of multiple serotypes) (1, 5, 14, 15).

Severe dengue illness is not currently treatable with medication, and the only form of assistance is supportive care. Unfortunately, the development of safe and fully protective dengue vaccines does not appear likely in the near future; therefore, early detection is crucial for success in disease management and epidemiological surveillance (3–5). The diagnosis of dengue virus infections is not based on clinical symptoms though, due to the symptoms of dengue being similar to those of many other diseases (16, 17). Definitive methods used in laboratories for the diagnosis of dengue fever include viral isolation, viral RNA detection, IgM/IgG antibody detection, and NS1 antigen detection (16–21). The sensitivity of the first two methods (viral isolation and viral RNA detection) is far greater when they are performed during viremia (within 5 days of the onset of fever), in which the serotype of DENV can be confirmed; however, these methods require special equipment and trained personnel available only in major medical centers. Furthermore, viral isolation is time-consuming and viral RNA detection is too expensive for all but the most advanced hospitals in developed countries (16, 22–24). IgM/IgG antibody detection (by enzyme-linked immunosorbent assay [ELISA] and lateral flow assay) is currently the method most commonly used to detect dengue diseases; however, it is inapplicable to the detection of DENVs in the early stages of infection and often produces false positives in cases where a patient has previously been infected with DENVs or other flaviviruses. Moreover, paired serum samples are required to confirm the diagnosis (19–21). NS1 antigen detection exploits the presence of dengue virus NS1, a highly conserved glycoprotein with a molecular weight of approximately 45 to 55 kDa. This antigen exists in monomer, dimer, and hexamer forms (25). Infected patients secrete large quantities of soluble NS1 (sNS1) into the bloodstream, such that the concentrations can reach up to 50 μg/ml (26, 27). sNS1 can remain in the blood for 9 days and persist for up to 18 days in some patients (28, 29), making NS1 an ideal biomarker during the early stages of illness, as it provides a wide window for dengue virus detection (25, 29). Dengue virus NS1 detection systems (ELISA and lateral flow assays) are commercially available from various manufacturers (30–34). However, none of the existing commercial kits has the capacity to identify the dengue serotype.

To overcome the limitations of the current commercial dengue virus NS1 detection systems, our primary objective in this study was to develop a sensitive NS1 antigen ELISA capable of detecting NS1 antigens and differentiating among the four dengue virus serotypes. We used one serotype-cross-reactive monoclonal antibody (MAb; as the capture antibody against NS1 protein) paired with one of four monoclonal antibodies (each of which was specific to NS1 of a particular serotype of dengue virus) to create our ELISA for DENV NS1 that can simultaneously detect the NS1 antigen and identify the dengue virus serotype. The clinical diagnostic benefits and utilities of our ELISA for DENV NS1 include the identification of patients at risk of severe dengue disease at the early stages of illness, because dengue virus serotypes can differ in virulence and epidemic capacity (35–37). Early detection also provides effective patient management, especially in areas where multiple serotypes cocirculate. As our ELISA for DENV NS1 is a simple and straightforward assay that has a high sensitivity and a high specificity in discriminating dengue virus serotypes, its application to clinical diagnosis and mass serotype screening is advantageous, even in regions where medical resources are limited and/or where multiple DENVs cocirculate.

## MATERIALS AND METHODS

### Viral strains.

Four dengue viral strains (DENV1 Hawaii, DENV2 16681, DENV3 H87, and DENV4 H241) were propagated in Aedes albopictus C6/36 cells in RPMI 1640 medium supplemented with 2% fetal bovine serum (FBS) and then incubated at 28°C until cytopathic effects were observed. As a control, Japanese encephalitis virus (JEV) strain SA14-14-2 was propagated in BHK-21 cells, and the Zika virus (ZIKV) ATCC VR 1843 strain was propagated in Vero cells that had been incubated in RPMI 1640 medium containing 2% FBS at 37°C for 2 to 3 days. (3) DENV and JEV titers were determined via plaque assays in BHK-21 cells. ZIKV titers were determined via plaque assays in Vero cells.

### Process of development of ELISA for DENV NS1.

**FIG 1:**
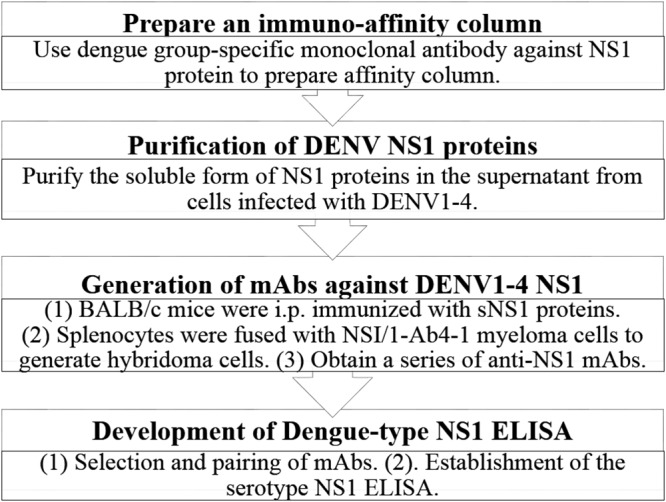
Procedures in development of ELISA for DENV NS1 (Dengue-type NS1 ELISA).

The full details and methods of the process of development of our ELISA for DENV NS1 can be found in Fig. 1 and in the supplemental material. Briefly, NS1 proteins were purified from cell culture supernatants of Vero cells infected with DENV1 Hawaii, DENV2 16681, DENV3 H87, and DENV4 H241 by immunoaffinity chromatography. BALB/c mice were initially immunized with immunoaffinity-purified NS1 proteins for the generation of hybridoma cells and the eventual selection of monoclonal antibodies against the four forms of NS1 protein. All experiments were performed using BALB/c mice purchased from the National Laboratory Animal Center and maintained at the Institute of Preventive Medicine’s animal housing facility. All animals were cared for in compliance with the guideline for the care and use of laboratory animals (2010, Taiwan, Republic of China), and experiments were reviewed and approved by the Institutional Animal Care and Use Committee or Panel of the Institute of Preventive Medicine (IACUC no. AN-104-12 and AN-105-05). Following a series of experiments for the selection of MAb pairs to assemble our ELISA for DENV NS1, the serotype-cross-reactive MAb that we named MAb53-1.1 was selected as the optimal capture antibody for pairing with four serotype-specific MAbs (named MAb12-4.1, MAb33-7.1, MAb43-1.3, and MAb22-1.5) as detection antibodies.

### Clinical samples.

A total of 146 clinical serum samples were used in this study. These included 55 samples from cases of confirmed dengue virus infection and 30 samples from nondengue cases (provided by the Centers for Disease Control, Department of Health, Taipei, Taiwan) as well as 61 samples from patients with suspected dengue virus infection collected from three hospitals from 2016 to 2018 (the Kaohsiung Armed Forces General Hospital, the Zuoying Branch of the Kaohsiung Armed Forces General Hospital, and the Tangshan Branch of the Kaohsiung Armed Forces General Hospital). All clinical serum samples were collected during the acute phase (1 to 7 days after the onset of illness) in Taiwan. Samples were tested using a serotype-specific one-step SYBR green I real-time reverse transcription (RT)-PCR, a dengue virus-specific IgM/IgG capture ELISA, the commercially available Platelia Dengue NS1 Ag ELISA kit (Bio-Rad, Marnes-la-Coquette, France), and our ELISA for DENV NS1. Negative results for non-dengue viral infections were obtained using a serotype-specific RT-PCR, the dengue virus-specific IgM/IgG capture ELISA, and the Platelia Dengue NS1 Ag ELISA. The study protocol was approved by the Kaohsiung Armed Forces General Hospital Institutional Review Board (IRB no. KAFGH 104-048). All patient clinical samples were deidentified, i.e., made anonymous, prior to analysis of the study data.

### Serotype-specific one-step SYBR green I real-time RT-PCR.

We performed the one-step SYBR green I real-time RT-PCR (QuantiTect SYBR green RT-PCR kit; Qiagen, Hilden, Germany) using four sets of serotype-specific primers in accordance with the methods previously described by Shu et al. (22). This involved the extraction of RNA from 140-μl blood samples using a QIAamp viral RNA minikit (Qiagen, Hilden, Germany) in accordance with the manufacturer’s instructions. RT-PCR assays were performed in a 25-μl reaction mixture containing 5 μl of sample RNA, 12.5 μl of RT-PCR master mix, each of the primer pairs, and 0.25 μl QuantiTect RT mix. The thermal profile for the RT-PCRs consisted of a 30-min RT step at 50°C and a 15-min *Taq* polymerase activation step at 95°C, followed by 45 cycles of PCR at 95°C for 15 s (denaturation), 55°C for 30 s (annealing), 72°C for 20 s (extension), and 77°C for 20 s (fluorescence detection). Melting curve analysis included a denaturing step at 95°C for 1 min, a drop in temperature to 68°C for 30 s, and then a gradual increase in temperature from 68°C to 95°C at a rate of 0.1°C/s, during which the fluorescence was continuously read.

### Dengue virus-specific IgM/IgG capture ELISA.

Each of the microplate wells was coated with 100 μl of 5 μg/ml of goat anti-human IgM (μ chain-specific) or anti-human IgG (γ chain-specific) antibodies in 0.1 M carbonate buffer (Na_2_CO_3_-NaOHCO_3_, pH 9.5) and left to stand at 4°C overnight. After washing and blocking, the wells were incubated with 100 μl of 1:100-diluted serum in phosphate-buffered saline (PBS)–Tween 20–1% bovine serum albumin (BSA)–5% normal rabbit serum and incubated at 37°C for 30 min. A cocktail (100 μl) containing pooled virus antigens (diluted to a concentration of 1:5) from the culture supernatant of Vero cells infected with DENV1 to DENV4 and MAb D56.3-alkaline phosphatase (1 μg/ml) was added to the wells, and the plate was allowed to stand at 37°C for 30 min. After a subsequent round of washing, enzyme activity was developed and the optical density (OD) at 405 nm (OD_405_)/OD_620_ was measured after a brief period of 30 min, in accordance with the methods previously described by Shu et al. (38).

### Platelia Dengue NS1 Ag ELISA.

DENV NS1 proteins were detected in clinical serum samples using the commercially available Platelia Dengue NS1 Ag ELISA kit (Bio-Rad, Marnes-la-Coquette, France) in accordance with the manufacturer’s instructions. Briefly, dilutions of positive-control, negative-control, calibration standard, and clinical samples were incubated in microwell plates with horseradish peroxidase (HRP)-conjugated MAbs at 37°C for 90 min. After washing the plates six times, 160 μl of tetramethylbenzidine was added to each well, whereupon the plates were incubated at room temperature away from the light for 30 min. The enzymatic reaction was stopped by adding 100 μl of stop solution, and OD_450_/OD_620_ measurements were obtained.

### Testing clinical serum samples using the ELISA for DENV NS1.

Microwell plates were coated with 100 μl of 10-μg/ml MAb53-1.1 and incubated at 4°C overnight. The wells were subsequently blocked using blocking buffer (PBS, 0.05% Tween, 5% skim milk), before undergoing incubation with 25 to 50 μl of serum diluted in blocking buffer at a 1:1 ratio at 37°C for 1 h. The plates were then washed four times using 0.05% PBS with Tween 20 and incubated with 100 μl of 0.8 μg/ml of serotype-specific MAb-HRP (MAb12-4.1, MAb33-7.1, MAb43-1.3, and MAb22-1.5) at 37°C for 1 h, before undergoing washing four additional times. Subsequent steps involved in the testing of clinical serum samples were in accordance with the methods described in the supplemental material (see “Development of Dengue-type NS1 ELISA”). Normal human serum samples (Sigma-Aldrich, Saint Louis, MO, USA) were used as a negative control. For each serotype, samples with values that exceeded the reference cutoff value (calculated as 2-fold the mean for the negative control) were considered positive by NS1 capture ELISA testing.

### Reproducibility.

A total of 57 DENV-positive serum samples (16, 17, 16, and 8 serum samples positive for DENV1 to DENV4, respectively) and 50 negative serum samples were tested using our ELISA for DENV NS1 by a second operator on a different day. Note that the volume of the 39 serum samples was insufficient to allow for repeat testing.

### Statistical analysis.

The sensitivity and specificity of serotyping by our ELISA for DENV NS1 were compared with those of serotyping by RT-PCR. The overall performances of our ELISA for DENV NS1 and the Platelia Dengue NS1 Ag ELISA were compared with the results from the sum of serotyping by RT-PCR and the dengue virus-specific IgM/IgG capture ELISA. The diagnostic accuracy, sensitivity, and specificity and the corresponding 95% confidence intervals (CI) for both ELISAs were determined using GraphPad Prism (version 6.0) software (GraphPad Software, San Diego, CA), with the significance level being set at a *P* value of <0.05.

## RESULTS

### Selection and characterization of anti-DENV NS1 MAbs.

In this study, a total of 136 hybridoma cell lines were generated to produce monoclonal antibodies for subsequent characterization. The reactivity of these MAbs with DENV1 to DENV4 was determined using ELISA and Western blot analysis. We confirmed the serotype specificity of 23 of these MAbs to the NS1 proteins of DENV (5 MAbs against DENV1 NS1, 5 MAbs against DENV2 NS1, 8 MAbs against DENV3 NS1, and 5 MAbs against DENV4 NS1; we also found that 4 of the 136 MAbs cross-reacted with the NS1 proteins of other DENVs). Confirmation was based on strong positive reactions observed when (i) indirect ELISA was performed using immunoaffinity-purified NS1 proteins as antigens and (ii) Western blot analysis was performed on lysates from C6/36 cells infected with DENV.

### Establishment of ELISA for DENV NS1.

**FIG 2:**
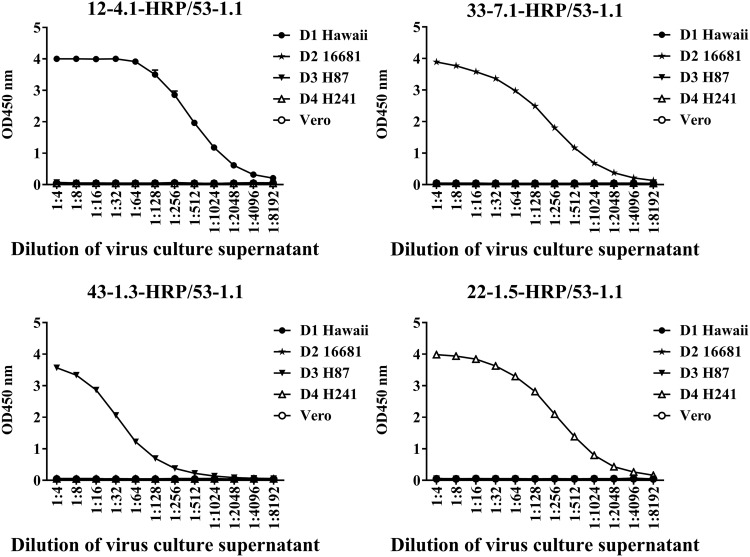
Sensitivity and specificity of HRP-conjugated serotype-specific antibodies paired with cross-reactive MAb53-1.1. Sensitivity was assessed by testing serial dilutions of supernatant from Vero cell cultures infected with DENV, whereas specificity was assessed in terms of the ability to differentiate among DENV serotypes: DENV1 (MAb12-4.1), DENV2 (MAb33-7.1), DENV3 (MAb43-1.3), and DENV4 (MAb22-1.5).

**FIG 3:**
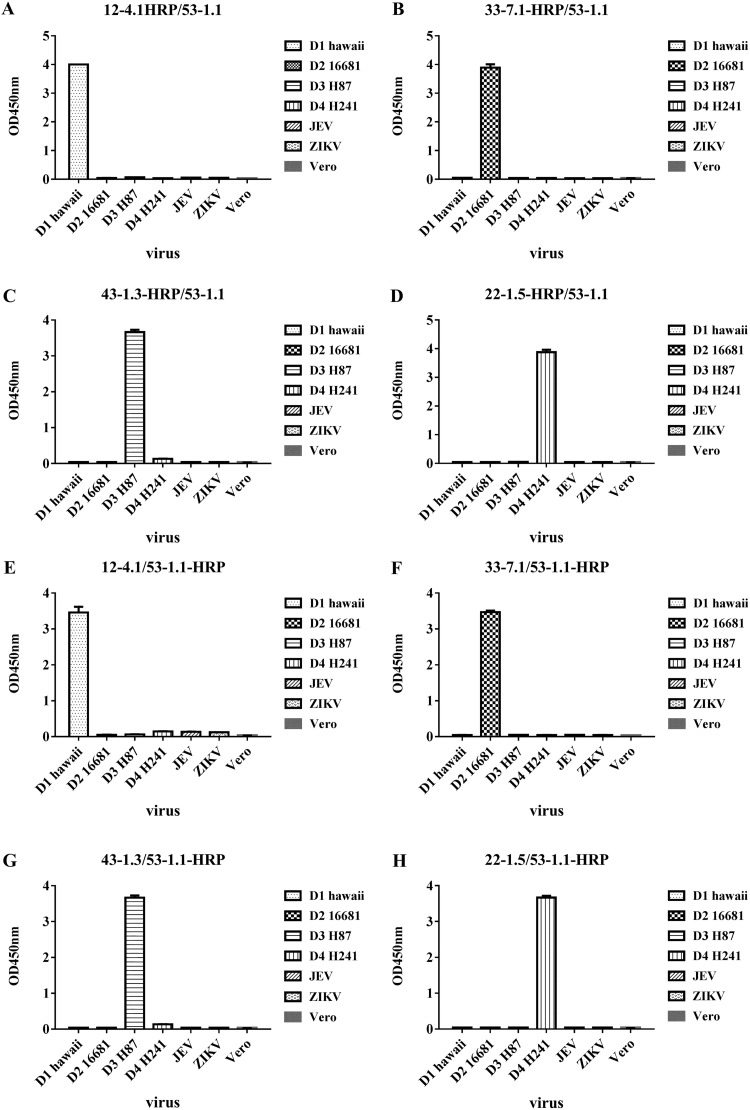
Orientation and specificity of sandwich ELISA for the detection of NS1 proteins in cell culture. In assessing specificity, cell culture supernatants from Vero cells infected with DENV1, DENV2, DENV3, DENV4, JEV, or ZIKV were used as NS1 antigen sources, and the supernatant from noninfected Vero cells was used as a negative control. The antibodies were paired as follows: serotype-specific MAbs (detection antibodies) were paired with cross-reactive (capture antibodies) (A to D), and serotype-specific MAbs (capture antibodies) were paired with cross-reactive MAbs (detection antibodies) (E to H).

Our ELISA for DENV NS1 was established by identifying the optimal MAb combinations, and the various combinations were analyzed in terms of sensitivity (via serial dilution of supernatant from Vero cell cultures infected with DENVs) and specificity (ability to differentiate among the DENV serotypes). As shown in Fig. 2, we selected four antibodies paired with MAb53-1.1 (a serotype-cross-reactive IgG2a heavy-chain MAb recognizing linear epitopes of the NS1 protein) that resulted in a high sensitivity and a high specificity to establish our ELISA for DENV NS1. The four MAbs presenting serotype-specific reactivity to DENVs that recognize the conformational epitopes of the NS1 protein were as follows: MAb12-4.1 for DENV1, MAb33-7.1 for DENV2, MAb43-1.3 for DENV3, and MAb22-1.5 for DENV4. The characteristics of the five MAbs used are listed in Table 1. We also tested which of the five antibodies were best suited as capture or detection antibodies. In one test, MAb53-1.1 acted as the solid-phase immobilized capture antibody and the other four serotype-specific MAbs (MAb12-4.1, MAb33-7.1, MAb43-1.3, and MAb22-1.5) were used as detection antibodies (Fig. 3A to D). In another test, four of the serotype-specific MAbs (MAb12-4.1, MAb33-7.1, MAb43-1.3, and MAb22-1.5) acted as the solid-phase immobilized capture antibodies, whereas MAb53-1.1 was used as the detection antibody (Fig. 3E to H). Our results revealed that all eight of the combinations provided strong specificity in differentiating among the four serotypes of DENV and presented no signs of reactivity with JEV or ZIKV NS1 (Fig. 3A to H). We subsequently selected MAb53-1.1 as the capture antibody and the serotype-specific antibodies MAb12-4.1, MAb33-7.1, MAb43-1.3, and MAb22-1.5 as the detection antibodies. We also determined that the optimal concentrations for the capture MAb and detection MAb were 1 μg/well and 0.08 μg/well, respectively.

**TABLE 1:** Characterization of reactions between MAb and NS1 proteins of DENV

Hybridoma	Isotype	Type of epitope	Reactivity of the four DENV serotypes by:								Specificity
			Western blotting^*a*^				ELISA^*b*^				
			DENV1	DENV2	DENV3	DENV4	DENV1	DENV2	DENV3	DENV4	
53-1.1	IgG2a(κ)	Linear	+	+	+	+	+	+	+	+	NS1
12-4.1	IgG1(κ)	Conformational	+	−	−	−	+	−	−	−	NS1
33-7.1	IgG1(κ)	Conformational	−	+	−	−	−	+	−	−	NS1
43-1.3	IgG2a(κ)	Conformational	−	−	+	−	−	−	+	−	NS1
22-1.5	IgG1(κ)	Conformational	−	−	−	+	−	−	−	+	NS1

a The lysates of C6/36 cells infected with different dengue virus serotypes were treated using SDS-PAGE sample buffer and gel electrophoresis, before being transferred to a nitrocellulose membrane and blotted with each MAb.

b Different NS1 antigens were immunoaffinity purified from the cell culture supernatants of Vero cells infected with the different serotypes of DENV. Microwell plates were coated with specific NS1 antigens and reacted with each MAb.

### Detection limits.

To determine the baseline detection limits of our ELISA for DENV NS1, we performed 2-fold serial dilutions of immunoaffinity-purified DENV NS1 proteins using blocking buffer, in which bovine serum albumin (BSA) was used to establish baselines for the assay. Samples with OD_450_ values at least double the OD_450_ of BSA were considered positive. Under this criterion, the linear portion of the standard curves of the four dengue virus serotype-specific ELISAs ranged from 3.906 to 125 ng/ml (see Fig. S1 in the supplemental material). The minimum detection levels of our ELISA for DENV NS1 were determined to be between 1 and 4 ng/ml, depending on the assay (sensitivities for DENV1 to DENV4 detection were, respectively, 1.953 ng/ml, 3.906 ng/ml, 3.906 ng/ml, and 0.977 ng/ml; Table 2). The assay for DENV4 was more sensitive than the assays for the other three serotypes. In contrast, the minimum detection levels achieved by the commercial Platelia Dengue NS1 Ag ELISA, which were determined at the same time using the same four purified NS1 proteins, were 3.906 ng/ml, 31.250 ng/ml, 0.977 ng/ml, and 7.813 ng/ml for DENV1 to DENV4, respectively (Table 2). This clearly demonstrates the superiority of our ELISA for the detection of DENV NS1 in terms of sensitivity.

**TABLE 2:** Detection limits of ELISA for DENV NS1 and Platelia Dengue NS1 Ag ELISA^*a*^

NS1 detection assay	Minimum detectable concn (ng/ml) of NS1 proteins from:			
	DENV1	DENV2	DENV3	DENV4
ELISA for DENV NS1	1.953	3.906	3.906	0.977
Platelia Dengue NS1 Ag ELISA	3.906	31.250	0.977	7.813

a The sensitivities of the ELISA for DENV NS1 and the commercial Platelia Dengue NS1 Ag ELISA for NS1 detection were determined. The NS1 protein of each serotype was immunoaffinity purified and serially diluted prior to analysis.

### Clinical tests.

We also evaluated the specificity and sensitivity of our ELISA for DENV NS1 using clinical serum samples. The analysis involved the collection of 146 clinical serum samples between 1 and 7 days after the onset of illness. The samples were analyzed using a serotype-specific one-step SYBR green I real-time RT-PCR, the dengue virus-specific IgM/IgG capture ELISA, the Platelia Dengue NS1 Ag ELISA, and our ELISA for DENV NS1. Following RT-PCR analysis, we found that 61 serum samples tested positive for DENV1 to DENV4 (17, 19, 16, and 9 serum samples were positive for DENV1 to DENV4, respectively; Table 3). Among the other serum samples, 5 that tested negative using RT-PCR went on to test positive following analysis with the dengue virus-specific IgM/IgG capture ELISA and the Platelia Dengue NS1 Ag ELISA, and our ELISA for DENV NS1 method subsequently identified these to be positive for DENV2 infection (Table 4). An additional 80 samples from non-dengue viral infections tested negative when using the serotyping RT-PCR, the dengue virus-specific IgM/IgG capture ELISA, and the Platelia Dengue NS1 Ag ELISA (Table 3). Among the 146 serum samples, 55 tested positive when the Platelia NS1 Ag ELISA was used, whereas our ELISA for DENV NS1 identified 56 positive samples (15, 23, 12, and 6 serum samples tested positive for DENV1 to DENV4, respectively; Table 3). By comparing these results with the ones obtained from the serotyping RT-PCR, the sensitivities of our ELISA for DENV NS1 were as follows: 88.2% (15/17), 94.7% (18/19), 75% (12/16), and 66.6% (6/9) for DENV1 to DENV4, respectively (Tables 4 and 5). Importantly, none of our four ELISAs showed any indication of cross-reactivity to any serotype following analysis, indicating a serotype specificity of 100%. Furthermore, our ELISA for DENV NS1 correctly identified all 80 serum samples from patients with non-dengue viral infections to be negative (Tables 4 and 5). These results clearly demonstrate the efficacy of our ELISA for DENV NS1 method for the detection of DENV NS1 and its ability to discriminate among serotypes of DENV in serum specimens, even during the acute phase of DENV infection, as early as day 1 after the onset of illness (Table 3). Despite the fact that the Platelia Dengue NS1 Ag ELISA and our ELISA for DENV NS1 achieved similar sensitivities (55 and 56 positive serum samples, respectively), we believe that our NS1 ELISA system is superior because it is able to differentiate the dengue virus serotypes.

**TABLE 3:** Analysis of acute-phase clinical serum samples by serotyping RT-PCR, IgM/IgG capture ELISA, Platelia Dengue NS1 Ag ELISA, and ELISA for DENV NS1

Day after onset of illness that serum was collected	Total no. of serum samples	No. of serum samples positive											
		RT-PCR serotyping (total *n* = 61)^*b*^				IgM/IgG capture ELISA^*c*^	Platelia dengue NS1 Ag ELISA (total *n* = 55)	Dengue viral infections^*d*^	Non-dengue viral infections^*e*^	ELISA for DENV NS1 (total *n* = 56)			
		DENV1	DENV2	DENV3	DENV4					DENV1	DENV2	DENV3	DENV4
1	45	6	4	4	4	0	14	18	27	5	4	4	3
2	46	4	7	8	1	2	19	22	24	3	9	6	0
3	21	3	1	1	2	0	4	7	14	3	1	0	2
4	12	2	3	2	1	0	8	8	4	2	3	1	1
5	7	0	2	0	0	1	2	3	4	0	2	0	0
6	6	1	1	1	0	1	4	4	2	1	2	1	0
7	1	0	0	0	0	0	0	0	1	0	0	0	0
Unknown^*a*^	8	1	1	0	1	1	4	4	4	1	2	0	0
													
Total	146	17	19	16	9	5	55	66	80	15	23	12	6

a No information pertaining to the serum collection date was available.

b Serotype-specific one-step SYBR green I-based RT-PCR.

c Negative results were obtained using RT-PCR; a positive result was obtained using a dengue virus-specific IgM/IgG capture ELISA and the Platelia Dengue NS1 Ag ELISA.

d Positive results were obtained using RT-PCR or a dengue virus-specific IgM/IgG capture ELISA and the Platelia Dengue NS1 Ag ELISA.

e Negative results were obtained using RT-PCR, a dengue virus-specific IgM/IgG capture ELISA, and the Platelia Dengue NS1 Ag ELISA.

**TABLE 4:** Performance of ELISA for DENV NS1 and Platelia Dengue NS1 ELISA for detection of NS1 in acute-phase sera

Virus	Total no. of serum samples	No. of serum samples positive by:				
		ELISA for DENV NS1 (*n* = 56)				Platelia Dengue NS1 Ag ELISA (*n* = 55)
		DENV1	D2ENV	DENV3	DENV4	
DENV1^*a*^	17	15	0	0	0	15
DENV2^*a*^	19	0	18	0	0	16
DENV3^*a*^	16	0	0	12	0	13
DENV4^*a*^	9	0	0	0	6	6
Any DENV^*b*^	5	0	5	0	0	5
Non-dengue virus^*c*^	80	0	0	0	0	0

a The serotype was determined using a serotype-specific one-step SYBR green I-based RT-PCR.

b Negative results were obtained using the serotype-specific RT-PCR; positive results were obtained using a dengue virus-specific IgM/IgG capture ELISA and the Platelia Dengue NS1 Ag ELISA.

c Negative results were obtained using the serotype-specific RT-PCR, the dengue virus-specific IgM/IgG capture ELISA, and the Platelia Dengue NS1 Ag ELISA.

**TABLE 5:** Serotype specificity and sensitivity of ELISA for DENV NS1 for the detection of NS1 in acute-phase sera

DENV serotype	No. of serum samples with the following results:					Serotype specificity (%)	Serotype sensitivity (%)
	Positive by RT-PCR	True positive	True negative	False positive	False negative		
DENV1	17	15	80	0	2	100 [80/(0 + 80) · 100]	88.2 [15/(15 + 2) · 100]
DENV2	19	18	80	0	1	100 [80/(0 + 80) · 100]	94.7 [18/(18 + 1) · 100]
DENV3	16	12	80	0	4	100 [80/(0 + 80) · 100]	75 [12/(12 + 4) · 100]
DENV4	9	6	80	0	3	100 [80/(0 + 80) · 100]	66.6 [6/(6 + 3) · 100]

Table 6 illustrates the overall diagnostic performances of our ELISA for DENV NS1 method and the Platelia Dengue NS1 Ag ELISA (of the 146 clinical serum samples, 66 serum samples were from patients with dengue viral infections and 80 serum samples were from patients with non-dengue viral infections). Our ELISA for DENV NS1 achieved a diagnostic accuracy of 93.15% (95% CI, 87.76 to 96.67%), with a specificity of 100% (95% CI, 95.49 to 100.0%), a sensitivity of 84.85% (95% CI, 73.9 to 92.49%), a positive predictive value of 100% (95% CI, 93.63% to 100%), and a negative predictive value of 88.89% (95% CI, 80.51 to 94.54%). In contrast, the Platelia Dengue NS1 Ag ELISA achieved a diagnostic accuracy of 92.47% (95% CI, 86.92 to 96.18%) with a specificity of 100% (95% CI, 95.49 to 100.00%) and a sensitivity of 83.33% (95% CI, 72.13 to 91.38%).

**TABLE 6:** Overall diagnostic accuracy and sensitivity of ELISA for DENV NS1 and Platelia dengue NS1 Ag ELISA^*a*^

ELISA	No. of serum samples positive for NS1	Sensitivity (% [95% CI])	Specificity (% [95% CI])	Accuracy (% [95% CI])	PPV (% [95% CI])	NPV (% [95% CI])
ELISA for DENV NS1	56	84.85 (73.9–92.49)	100 (95.49–100)	93.15 (87.76–96.67)	100 (93.63–100)	88.89 (80.51–94.54)
Platelia dengue NS1 Ag ELISA	55	83.33 (72.13–91.38)	100 (95.49–100)	92.47 (86.92–96.18)	100 (93.51–100)	87.91 (79.4–93.81)

a True status was determined for 66 patients with dengue virus infections and 80 patients with non-dengue virus infections. PPV, positive predictive value; NPV, negative predictive value.

### Reproducibility.

Clinical serum samples were retested with our ELISA for DENV NS1 by a second operator on a different day. The tests were performed using the original serum samples. Specifically, we tested a total of 57 samples that were positive for DENV (16, 17, 16, and 8 samples positive for DENV1 to DENV4, respectively) and 50 samples that were negative for DENV. The results from this second round of testing (107 serum samples; 39 samples could not be retested due to insufficient volumes of serum) were 100% consistent with those of the initial testing for DENV1 to DENV4, and all 50 negative serum samples again produced a negative result, thereby illustrating the reproducibility of our ELISA for DENV NS1.

## DISCUSSION

Dengue virus infections range from asymptomatic or mild to severe or life-threatening (1). The fact that the appropriate detection method varies with the stage of the disease makes it very difficult to secure a reliable clinical diagnosis (17, 19, 21). Large-scale epidemics often occur in areas with insufficient medical resources to treat every patient. In such cases, it is essential that medical staff have access to reliable detection methods capable of identifying the patients who are the most likely to progress to a more severe form of the disease.

The capture ELISA and the lateral flow assay for DENV NS1 are easy to use, highly specific, and inexpensive, and they possess the ability to detect dengue virus NS1 proteins during the early stages of infection. The NS1 ELISA and NS1 lateral flow assay have gradually become the standard for routine clinical assessments, despite their inability to differentiate a primary from secondary dengue virus infection. At present, no existing dengue virus NS1 test kit is able to differentiate among the various dengue virus serotypes. Numerous researchers have tried addressing this issue by developing antigen detection tests for dengue virus infections, generating methods for the creation of anti-DENV NS1 monoclonal antibodies (MAb), and devising tests for dengue virus serotyping. In 2011, Ding et al. (39) used a prokaryotic expression system to express recombinant NS1 proteins to immunize mice; however, the expressed NS1 was not in its native form and lacked protein glycosylation. In addition, their serotyping ELISAs were developed by pairing four serotype-specific capture MAbs with four other serotype-specific detection MAbs. In the same year, Puttikhunt et al. (40) used dengue virus-infected PS clone D cells or immunoaffinity-purified NS1 to immunize mice and subsequently developed an NS1 serotyping ELISA that employed a single flavivirus-cross-reactive IgM anti-NS1 MAb to capture NS1 antigens of all four serotypes, which were then detected using four anti-NS1 MAbs. The antigen-antibody complexes were subsequently detected using goat anti-mouse IgG conjugated with HRP. In 2017, Lebani et al. (41) isolated dengue virus serotype-specific anti-NS1 monoclonal antibodies using phage display biopanning. This involved selecting four serotype-specific MAbs for conjugation with different fluorescent microspheres, which were then paired with a DENV-cross-reactive MAb to enable the detection of DENV NS1. The use of multiplexed fluorescent microsphere beads, however, requires expensive machinery for detection. The following year, Röltgen et al. (42) used HEK cell surface-expressed NS1 to immunize mice for the generation of anti-DENV NS1 MAbs. They achieved serotyping by pairing four anti-NS1 MAbs as capture antibodies with four biotinylated anti-NS1 MAbs to measure complexes with HRP-labeled streptavidin.

Our study overcomes many of the limitations of the above-mentioned tests. The soluble NS1 proteins from the supernatant of Vero cells infected with DENV1 to DENV4 were purified in their authentic, native form with proper glycosylation. The native epitopes within our NS1 proteins enhance the ability of our generated monoclonal antibodies to recognize NS1 proteins in patient serum. We selected a single cross-reactive MAb as the capture antibody that was paired with one of the four serotype-specific MAbs-HRP to create our ELISA for DENV NS1. As a result, we established a serotyping assay using only five antibodies without the need for expensive equipment for detection.

The significance of the results in our study are outlined in the following. (i) Our ELISA for DENV NS1 was sensitive, with the lowest detectable concentration of an individual soluble NS1 being 1 to 4 ng/ml. (ii) The assay was specific, as our ELISA for DENV NS1 was able to discriminate the four dengue virus serotypes with no mutual cross-reactivity and no cross-reactivity with Japanese encephalitis virus or Zika virus (determined using supernatants of Vero cell cultures infected with DENV serotypes 1 to 4, JEV, and ZIKV). (iii) We evaluated the sensitivity and accuracy of our ELISA for DENV NS1 by applying it to clinical serum samples from 146 patients presenting with fever (66 patients with confirmed dengue viral infections and 80 patients with non-dengue viral infections). Our ELISA generated no false positives for any of the 80 samples from patients with non-dengue viral infections; i.e., the specificity was 100%. The overall detection rate of the test was 84.85%. With a comparison of the cases positive by the serotyping RT-PCR, the sensitivity of each type of ELISA was as follows: 88.2% (15/17), 94.7% (18/19), 75% (12/16), and 66.6% (6/9) for DENV1 to DENV4, respectively. In other words, our ELISA for DENV NS1 was able to identify the specific serotype of the dengue virus in samples from infected patients with a high degree of specificity and no cross-reactivity among serotypes. (iv) Five of the clinical samples examined in this study presented negative results when analyzed using the serotype RT-PCR test but were positive following analysis by the dengue IgM/IgG test and Platelia Dengue NS1 Ag ELISA kit. Our ELISA for DENV NS1 then determined that all five of these samples were positive for DENV2, even though dengue virus viremia may be lower in acute-phase serum samples; i.e., our system was still able to detect the low levels of NS1 antigen and correctly identify the serotype.

While there was no cross-reactivity with the NS1 proteins of Japanese encephalitis virus or Zika virus derived from the supernatant from cells in culture, our future studies could include the analysis of patient sera infected with viruses other than DENV, such as Japanese encephalitis virus, Zika virus, or West Nile virus. At present, there are no West Nile cases and only a few imported Zika cases in Taiwan. This is a limitation of our study. Moreover, our ELISA for DENV NS1 could be a tool to determine the number and identity of DENV serotypes during situations of DENV coinfection.

In Taiwan, there have been reports of local dengue outbreaks caused by different serotypes and genotypes of DENV as well as by the cocirculation of more than one DENV serotype at any given time (43–46). The Centers for Disease Control of Taiwan confirmed 15,732 cases of dengue fever in 2014 and 43,784 cases in 2015 (largely in southern Taiwan). DENV1 was the predominant strain in 2014 and persisted until 2015, whereupon DENV2 became the predominant strain. A larger number of deaths than in any other previous outbreak resulted from dengue hemorrhagic fever and dengue shock syndrome during the 2015 epidemic (47). Taiwan is located in a subtropical region, which is ideally suited to the spread of different DENV serotypes brought in by travelers returning from regions in tropical Southeast Asia where dengue is endemic. Indeed, this situation has already occurred, as manifested by the presence of Aedes aegypti mosquitoes in the southern part of the island (45, 48).

The severity of dengue disease is associated with the age and immune status of infected individuals as well as the presence of chronic comorbidities (49, 50). Infection with any serotype results in lifelong immunity to that serotype; however, the antibodies which are generated in response to one serotype only partially neutralize other serotypes. In fact, an initial DENV infection increases the likelihood that a second DENV infection with another serotype will develop into severe disease (11, 13). It is worth noting that the chance of a second DENV infection developing into severe dengue disease is also associated with the time interval between the two infections (51) and that some infection sequences are more likely to develop into a severe disease than other infection sequences (52–54). Although cases in which the first infection develops into severe dengue disease have been recorded, these cases are rare. The second infection typically presents the greatest danger. Interestingly, a third or fourth DENV infection usually presents as only a mild or asymptomatic infection (55, 56). Nonetheless, in a number of regions around areas of endemicity, multiple types of dengue virus persist, increasing the risk and likelihood that individuals who live in or travel to these regions will develop severe dengue disease.

During the early period of infection (i.e., within the first 5 days), performing viral isolation and viral RNA detection testing which is capable of detecting and determining the type of dengue virus responsible for infection not only is expensive but also requires special equipment and trained personnel. Therefore, the accurate and prompt diagnosis and treatment of patients in regions with limited resources are difficult. Our ELISA for DENV NS1 is capable of detecting DENV NS1 antigens rapidly in the early stage and identifying the specific serotype of dengue virus that is responsible for the infection, thereby overcoming the limitations of current commercial NS1 diagnostic tests. We believe that our ELISA for DENV NS1 could be especially beneficial in regions where medical resources are limited and where multiple DENVs cocirculate. Moreover, this test can be easily incorporated into routine clinical diagnostic procedures to facilitate mass serotyping during emerging epidemics to identify patients at risk of severe dengue disease. Our ELISA could also be used in retrospective research to elucidate the distributions of various DENVs as well as the factors that contribute to severe dengue disease. Finally, the implementation of our ELISA for DENV NS1 could improve epidemiological surveillance, outbreak monitoring, clinical management, and knowledge of the circulating serotypes of this disease. The next step in this project involves the development of an immunochromatographic (ICT) rapid test for use in point-of-care testing to facilitate the prevention of severe dengue disease.

## Supplementary Material

Supplemental file 1
