# Supplementary material for: Development of an Enzyme-Linked Immunosorbent Assay for Rapid Detection of Dengue Virus (DENV) NS1 and Differentiation of DENV Serotypes during Early Infection
Source: J Clin Microbiol. 2019 Jun 25;57(7):e00221-19. doi: 10.1128/JCM.00221-19 (PMC6595446; doi:10.1128/JCM.00221-19)
Supplement: Supplemental file 1 [file JCM.00221-19-s0001.pdf]

Materials and methods

Preparation of NS1 proteins. Cell culture supernatants of Vero cells infected with the four viruses（DENV1 Hawaii, DENV2 16681, DENV3 H87, and DENV4 H241) were harvested after 5 d and inactivated by UV irradiation. To purify the soluble form of NS1 proteins (for each of the dengue serotypes) in the supernatant from each cell culture, we employed immunoaffinity chromatography columns in accordance with the manufacturer's instructions. This involved the use of anti-NS1 monoclonal antibody D8-1 (flavivirus group-specific against NS1 proteins generated in our lab)(1), which had been immobilized onto a HiTrap™ NHS-activated HP column (GE Healthcare, Uppsala, Sweden). The column was equilibrated using five volumes of PBS before viral cell culture supernatants were passed over the immunoaffinity column to wash out unbound proteins using ten volumes of PBS. Bound NS1 was eluted using glycine solution (pH 2.8) before being buffered to neutral pH. The purified DENV NS1 proteins were then detected using commassie blue stained SDS-PAGE and Western blot analysis (in which mAb D8-1 was the primary antibody). The concentrations of NS1 proteins were measured using a Pierce BCA protein assay kit (Thermo Fisher Scientific, Illinois, USA).

Generation and characterization of serotype-specific mAbs against

DENV1-4 NS1. All experiments were performed using BALB/c mice purchased from the National Laboratory Animal Center and maintained at the Institute of Preventative Medicine’s animal housing facility. All Animals were cared for in compliance with the Guideline for the Care and Use of Laboratory Animals (2010, Taiwan, R.O.C.) and experiments were reviewed and approved by the Institutional Animal Care and Use Committee or Panel of Institute of Preventive Medicine (IACUC no. AN-104-12, AN-105-05). For the first inoculation, four-week-old BALB/c mice were i.p. immunized with 15 μg of immunoaffinity-purified NS1 proteins in complete Freund’s adjuvant. The mice were then administered subsequent immunization boosts against their respective serotypes using 15 μg of NS1 proteins in incomplete Freund’s adjuvant. DENV antisera were obtained from the mice after consecutive challenges. Briefly, the spleens of immunized mice were removed and splenocytes were fused with NSI/1-Ab4-1 myeloma cells to generate hybridoma cells, which were selected in accordance with the standard procedures outlined by Kohler and Milstein (1975)(2). The fused cells were washed twice with RPMI and then mixed with 1 ml 50% (w/v) PEG 1500 (Roche, Penzberg, Germany) (which was gradually added over a period of 1 min under gentle stirring) in a 15 ml conical tube. The mixture was then diluted twice through the slow (1 min) addition of 1 ml RPMI, followed by the slow addition

(2 min) of 8 ml serum-free RPMI. The mixture was subsequently centrifuged at 400g for 5 min. The fused cell pellet was re-suspended in RPMI supplemented with 20% FBS, HAT medium (Life technologies, Burlington, ONT Canada) and HFCS solution (Roche, Mannhein, Germany), and finally, the resuspension mixture was distributed in 96-well plates (200 µl per well). Hybridoma cell lines that secreted specific antibodies against NS1 were identified via indirect ELISA (using purified DENV NS1 as the coating antigen for each serotype). Single clone cells were generated by limiting dilution. Western blotting analysis of lysates from C6/36 cells infected with DENVs was performed to determine (a) the specificity of anti-NS1 mAbs and (b) whether the epitopes recognized by the antibodies were conformational or linear. The mAbs were isotyped using a commercial mouse monoclonal antibody isotyping kit (IsoStrip™, Roche, Mannheim, Germany). The hybridoma cells were i.p. injected into pristane-primed BALB/c mice to induce ascitic fluid production. The mAbs were then purified from ascitic fluids using a protein G-sepharose column (HiTrap protein G, GE Healthcare, Uppsala, Sweden) in accordance with the manufacturer's instructions.

HRP conjugation. To conjugate mAbs with HRP (Innova Biosciences, Cambridge, UK), 100 µg of HRP and a 20 µl aliquot of modifier reagent were mixed with 200 µl of 1 mg/ml mAb. After incubating the mixture for 3 h at room temperature (20~25℃),

the reaction was stopped using a 20 µl aliquot of quencher. Following incubation for an additional 30 min at room temperature, 260 µl of glycerol was added, and the final solution was stored at -20°C.The final concentration of mAb-HRP was 400 µg/ml.

Development of Dengue-type NS1 ELISA. The development of Dengue-type NS1 ELISA involved selecting several serotype-specific and highly reactive mAbs based on the characteristics of anti-NS1 mAbs. Our objective was to determine (1) the sensitivity and specificity of each combination by testing HRP conjugated serotype-specific antibodies via pairing with serotype-cross-reactive mAb53-1.1 in a sandwich capture ELISA format, and (2) whether serotype-specific mAbs and serotype-cross-reactive mAb should be used as capture or detection antibodies. The tests were conducted using (a) serotype-specific mAbs as a detection antibody and cross-reactive mAb as a capture antibody or (b) serotype-specific mAbs as a capture antibody and cross-reactive mAb as a detection antibody. The serotype-cross-reactive mAb53-1.1 was selected as the optimal capture antibody for pairing with the four serotype-specific mAbs (mAb12-4.1, mAb33-7.1, mAb43-1.3, and mAb22-1.5) as detection antibodies for the assembly of Dengue-type NS1 ELISA. We determined the optimal experimental conditions for the coating and dilution of mAb-HRP using checkerboard titration. Briefly, 96-well plates (Nunc Immuno Maxisorp, Thermo,

Roskilde, Denmark) were coated with 100 µl of 10 µg /ml of mAb53-1.1 and incubated overnight at 4°C. The wells were subsequently blocked using blocking buffer (PBS, 0.05% Tween, 5% skim milk) at 37°C for 1 h and washed with wash buffer (PBS, 0.05% Tween). Viral culture supernatant or NS1 proteins were then serially diluted using blocking buffer and incubated at 37°C for 1 h. The plates were then washed before adding 100 µl of 0.8 µg/ml of serotype-specific mAb-HRP to undergo incubation again at 37°C for 1 h. Finally, the microwell plates were washed once again before adding 100 µl of TMB reagent to undergo incubation again at room temperature for 10 min. The reaction was stopped using 1 N sulfuric acid, whereupon the absorbance was read at 450 nm using a microplate autoreader.

Supplemental References

1. Chen L-K, Liao C-L, Lin C-G, Lai S-C, Liu C-I, Ma S-H, Huang Y-Y, Lin Y-L. 1996. Persistence of Japanese Encephalitis Virus Is Associated with Abnormal Expression of the Nonstructural Protein NS1 in Host Cells. Virology 217:220-229.

2. KÖ Hler G, Milstein C. 1975. Continuous cultures of fused cells secreting antibody of predefined specificity. Nature 256:495.

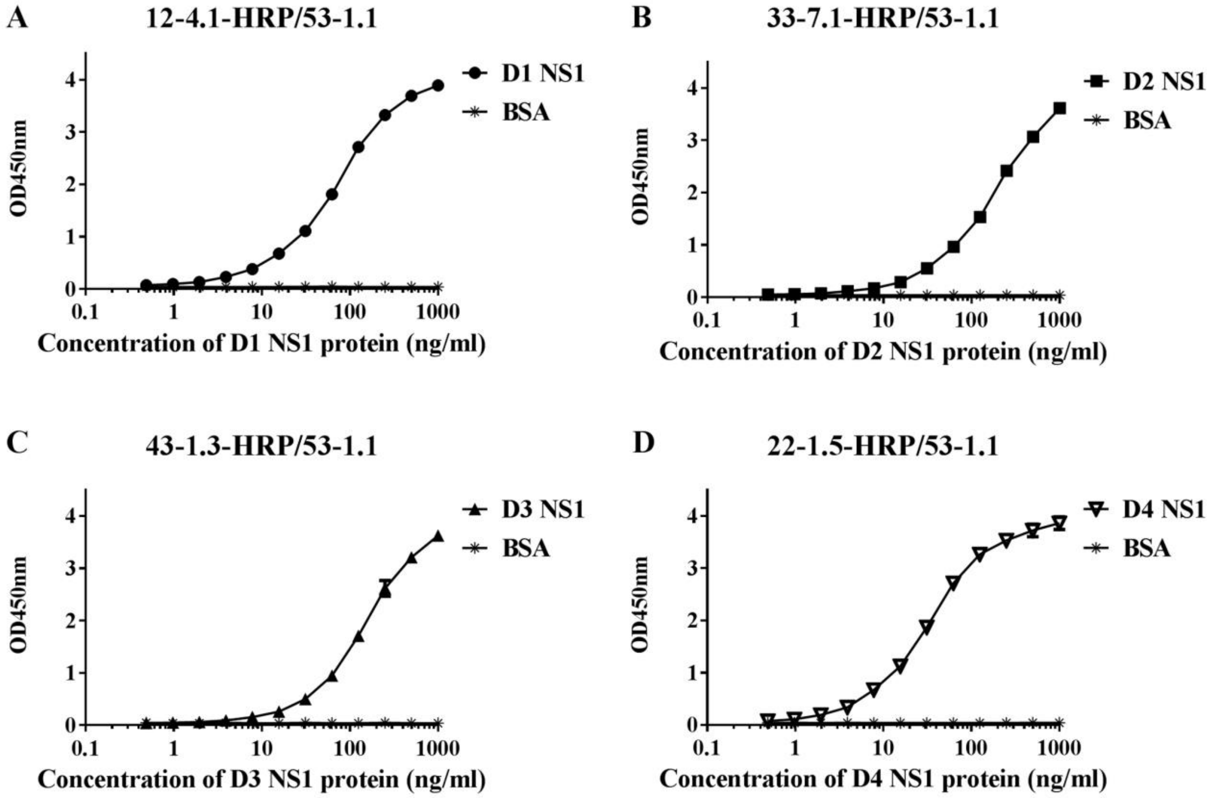

Figure S1. Standard curves illustrating the reactions between Dengue type NS1 ELISA and purified DENV NS1 proteins. The immunoaffinity-purified NS1 of each DENV serotype was serially diluted 2-fold and analyzed (A-D); BSA was used to establish a baseline for the assays. The minimum immunoaffinity-purified NS1 protein detection using these assays resulted in an OD450 value 2-fold higher than BSA. Each data point represents the mean ± SD of 3 replicated tests.
